# The Relative Weight of Temporal Envelope Cues in Different Frequency Regions for Mandarin Sentence Recognition

**DOI:** 10.1155/2017/7416727

**Published:** 2017-01-19

**Authors:** Yang Guo, Yuanyuan Sun, Yanmei Feng, Yujun Zhang, Shankai Yin

**Affiliations:** Department of Otolaryngology Head and Neck Surgery, Shanghai Jiao Tong University Affiliated Sixth People's Hospital, No. 600, Yishan Road, Xuhui District, Shanghai 200233, China

## Abstract

Acoustic temporal envelope (E) cues containing speech information are distributed across the frequency spectrum. To investigate the relative weight of E cues in different frequency regions for Mandarin sentence recognition, E information was extracted from 30 contiguous bands across the range of 80–7,562 Hz using Hilbert decomposition and then allocated to five frequency regions. Recognition scores were obtained with acoustic E cues from 1 or 2 random regions from 40 normal-hearing listeners. While the recognition scores ranged from 8.2% to 16.3% when E information from only one region was available, the scores ranged from 57.9% to 87.7% when E information from two frequency regions was presented, suggesting a synergistic effect among the temporal E cues in different frequency regions. Next, the relative contributions of the E information from the five frequency regions to sentence perception were computed using a least-squares approach. The results demonstrated that, for Mandarin Chinese, a tonal language, the temporal E cues of Frequency Region 1 (80–502 Hz) and Region 3 (1,022–1,913 Hz) contributed more to the intelligence of sentence recognition than other regions, particularly the region of 80–502 Hz, which contained fundamental frequency (*F*_0_) information.

## 1. Introduction

Speech is an indispensable process for communicating in everyday life; it is transmitted through the cochlea to the brain and then becomes understood. The cochlea is commonly referred to as a series of overlapping auditory filters that divide the normal frequency range of speech into narrow bands, with center frequencies corresponding to specific positions on the basilar membrane [1]. As the high-frequency sound causes maximum displacement of the basilar membrane near the base, the basilar membrane close to the apex vibrates strongest in response to the low-frequency sound. The speech signal within a narrow band is a compound signal consisting of two different kinds of information: the slowly varying amplitude, known as the temporal envelope (E), and rapid variations with rates close to the central frequency of the band, called the temporal fine structure (TFS) [2–4]. Acoustic E cues can provide sufficient information for nearly perfect speech recognition in a quiet environment, while the TFS is needed for a noisy background and for pitch and tonal recognition [3, 5, 6].

The redundant nature of speech, based on spectral and temporal cues, guarantees the intelligence of speech even under temporally and spectrally degraded conditions. Under these conditions, listeners use different strategies to make comprehension possible, such as temporal [7, 8] and spectral integration [9–11]. To understand the relative importance of the different spectral regions, much effort has been made over the years.

By changing the location of the spectral “hole” in the tonotopic representation of the cochlea in an orderly manner, Shannon et al. [12] suggested that a hole in the apical region was more detrimental to speech perception using temporal E information than a hole in the basal or middle regions. Ardoint and Lorenzi [3] adopted high-pass and low-pass method to show that the temporal E information in frequency regions of 1-2 kHz conveys important phonetic cues, while the synergistic effect [13] across frequency regions was not considered.

Taking the synergistic effect across frequency regions into account, Apoux and Bacon [14] used both the hole and the correlational methods to investigate the relative weight of temporal E information across spectral regions. However, they consistently found that temporal E cues contained in the highest frequency region (>3.5 kHz) were more important in a noisy environment. Subsequently, another recognition task with bandpass-filtered speech was conducted to evaluate the ability to use temporal E in different frequency regions of English [15]. The recognition scores of consonants were measured with only 1 frequency region or 2 disjointed or 2 adjacent regions. The performance increased as the region-center frequency increased consistently for both the processed single region and pairs of regions in a quiet environment, showing that E cues in higher frequency regions (1.8–7.3 kHz) contributed most to consonant recognition [15].

As mentioned above, most reported studies have explored the features of English, a nontonal language, while limited attention has been paid to Mandarin, a tonal language spoken by many people. Luo et al. [16, 17] showed that periodic fluctuation cues (50–500 Hz) in the highest frequency region (3043–6000 Hz) contributed the most to Mandarin tone recognition, while vowel recognition was not significantly affected by the availability of periodic fluctuation cues. For recognition of Mandarin sentence, however, little is known about the ability to use temporal E cues in different frequency regions and to combine the temporal E from various frequency regions. As a tonal language, the same phoneme with different tones has various meanings. For example, the syllable /ma/ can have different meanings depending on the *F*_0_ contours. Additionally, Mandarin-speaking listeners rely more on *F*_0_ variations to discriminate Thai lexical tones than do French-speaking listeners [18]. It has been established that changes in fundamental frequency (*F*_0_) play essential roles in tone identification [19, 20], which, in turn, contribute to Mandarin sentence recognition [17, 21].

Fogerty [22] suggested that acoustic TFS cues in the middle-frequency region (528–1,941 Hz) weigh most for English recognition while those in the low-frequency (80–528 Hz) and high-frequency (1,941–6,400 Hz) regions were much less important [22]. However, the findings from our previous study indicated that the acoustic TFS cues in the low-frequency region contributed more to Mandarin sentence recognition than English. For Mandarin, the relative weight of the acoustic TFS in the low-frequency region (~0.4) was slightly lower than that of the middle-frequency region (~0.5) [10].

Considering these apparent differences in the TFS weight distribution between English and Mandarin and the contribution of *F*_0_ to tone recognition, it is possible that the frequency-weighting functions of temporal E for Mandarin differ from those for English. The goal of this study was to investigate the relative weight of temporal E in different frequency regions for Mandarin sentence recognition in a quiet environment.

## 2. Materials and Methods

### 2.1. Participants

In total, 40 normal-hearing listeners (20 males, 20 females) were recruited in this study. Their ages ranged from 21 to 28 (average = 24.9) years. All subjects were native Mandarin speakers with no reported history of ear disease or hearing difficulty. All subjects were recruited from graduates of Shanghai Jiao Tong University and were tested at Shanghai Jiao Tong University Affiliated Sixth People's Hospital. In all participants, audiometric thresholds were at the ≤25 dB hearing level (HL), bilaterally, at octave intervals from 0.25 to 8 kHz. Pure-tone audiometric thresholds were recorded with a GSI-61 audiometer (Grason-Stadler, Madison, WI) using standard audiometric procedures [23]. No subject had any preceding exposure to the sentence materials.

This study was approved by the Ethics Committee of Shanghai Jiao Tong University Affiliated Sixth People's Hospital. Signed consent forms were obtained from all participants before testing, and they were compensated on an hourly basis for their participation.

### 2.2. Signal Processing

The Mandarin version of the hearing in noise test (MHINT), provided by the House Ear Institute, was used as original material, which was recorded digitally by a male speaker [24]. The MHINT materials consist of 12 lists, each comprising 20 sentences. With each sentence containing 10 key words, there are 240 key words in one list.

The sentences were filtered into 30 contiguous frequency bands using zero-phase, third-order Butterworth filters (36 dB/oct slopes), ranging from 80 to 7,562 Hz. Each band was an equivalent rectangular bandwidth (ERB_N_) for normal-hearing listeners, simulating the frequency selectivity of the normal auditory system [25]. E information was extracted from each band using the Hilbert decomposition and low-pass filter at 64 Hz using third-order Butterworth filters. Then E was used to modulate the amplitude of a white noise. The envelope-modulated noise was bandpass-filtered using the same filter parameters as before, after which the modulated noise bands were summed across frequency bands to produce the frequency regions of acoustic E cues as follows: Bands 1–8, 9–13, 14–18, 19–24, and 25–30 were summed to form Frequency Regions 1–5, respectively (Table 1).

To investigate the role of the frequency regions in sentence recognition, the acoustic E information from 1 frequency region (5 conditions), 2 adjacent frequency regions (4 conditions), 2 nonadjacent frequency regions (6 conditions), and all frequency regions (1 condition) was presented to subjects. To prevent the possible use of information from the transition bands [4, 26], frequency regions containing sentence E information were combined with complementary frequency regions containing noise masker that was presented at a speech-to-noise ratio (SNR) of +16 dB. As with the preparation of the frequency regions of the sentence E cues, the white noise was filtered into 30 contiguous frequency bands and summed to produce the frequency regions of noise. For example, the condition of “Region 1” means that the stimulus presented to the listeners consisted of sentence E information from Frequency Region 1 and noise from the other frequency regions (Regions 2–5). Similarly “Region 1 + 2” refers to a stimulus consisting of acoustic E information from Frequency Regions 1 and 2 and noise from the rest of the frequency regions (Regions 3–5). The “Full Region” stimulus consisted of E from all five frequency regions (Regions 1–5) with no added noise.

As there are 12 lists in the MHINT materials, there were 16 experimental conditions to be tested. The same list was not used in two different test conditions on one subject to avoid any learning effect. Thus, the 16 test conditions were divided into two groups. Group 1 completed 5 conditions with 1 frequency region, 4 conditions with 2 adjacent frequency regions, and 1 condition with the full frequency regions. Group 2 completed 4 conditions with 2 adjacent frequency regions and 6 conditions with 2 nonadjacent frequency regions. Thus, there were 10 conditions in each group, and the 4 experimental conditions with 2 adjacent frequency regions in the 2 groups were the same. Accordingly, there were 10 lists in each group of MHINT materials.

### 2.3. Procedures

The 40 participants were divided randomly and equally into groups 1 and 2, each comprising 10 males and 10 females. The participants were tested individually in a double-walled, sound-attenuated booth. Stimuli were presented binaurally to the participants through Sennheiser HD 205 II circumaural headphones at the most comfortable level for each subject, usually at 65 dBSPL. Each key word in a sentence was scored as correct or incorrect, and the performances were expressed as the percentage of correct words for the different conditions.

About 30 min of practice was provided prior to the formal test. The practice stimuli consisted of 40 sentences (two lists) of MHINT materials and were first presented under “Full Region” conditions and then processed in the same manner as the experimental stimuli. Feedback was provided during practice. To familiarize the participants with the processed stimuli, they could repeat a sentence as many times as they wished before moving on to the next sentence until their performance reached a plateau.

In the formal tests, the participants were permitted to listen to the sentence as many times as they wished. All 10 conditions, corresponding to 10 lists of MHINT materials, were presented in a random order across subjects to avoid any order effect. Participants were instructed to repeat the sentences as accurately as possible and were encouraged to guess if uncertain of the words in a sentence. No feedback was given during the test period. The subject could take a break whenever needed. The total duration of testing was ~2 h for each listener.

## 3. Results

### 3.1. Percent-Correct Scores for Sentence Recognition across Conditions Using Temporal E

**Figure 1:**
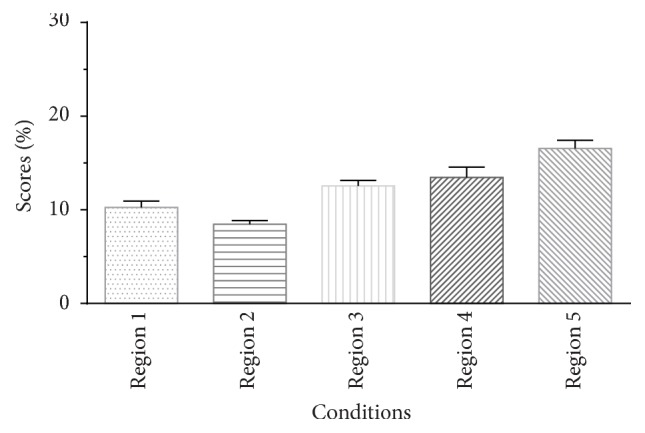
Averaged percent-correct scores for sentence recognition using acoustic temporal envelope as a function of condition in Group 1. The error bars indicate standard errors.

**Figure 2:**
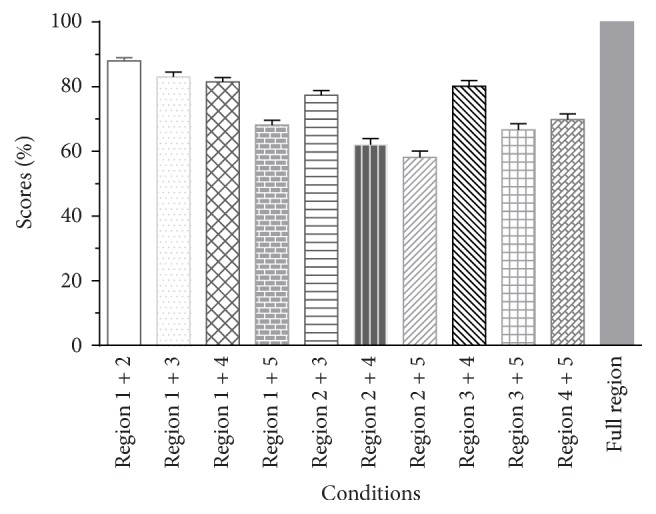
Averaged percent-correct scores for sentence recognition using acoustic temporal envelope as a function of conditions in Group 2 and the condition with a full frequency region in Group 1. The error bars indicate standard errors.

The mean percent-correct sentence recognition scores with acoustic E from one frequency region obtained from Group 1 are shown in Figure 1. The scores range from ~8.2% to ~16.3%, with the Region 5 condition scores being highest and the Region 2 scores being the lowest. Indeed, the listeners could not understand the meaning of the sentences under such adverse conditions. However, the intelligibility of sentences using temporal E approached perfect when all five regions were presented to the listener simultaneously (Figure 2). The data were transformed to rationalized arcsine units (RAU) for the purposes of statistical analyses [27]. A one-way repeated-measures analysis of variance (ANOVA) was used for the results from different conditions with one frequency region, showing a significant main effect of condition on sentence recognition (*F*(4,76) = 21.781, *p* < 0.001). The post hoc analysis (Tukey's test) revealed that the scores differed significantly between any two conditions (*p* < 0.05), except for the scores obtained from the Region 3 and Region 4 conditions.

As the conditions with two adjacent regions were the same in both subject groups, we compared the performance of these conditions in the two groups first (Table 2). Independent samples *t*-tests showed that the percent-correct score differences obtained from the same conditions in two groups were not significant (all *p* > 0.05). Therefore, the data obtained from the two groups were combined to calculate the relative weights of the five frequency regions.

As shown in Figure 2, under all conditions with two frequency regions, the score was >55%, and the Region 1 + 2 condition scores were the highest, ~87.7%, while Region 2 + 5 scores were the lowest, ~57.9%. Generally, the intelligence scores for conditions with two frequency regions tended to decrease as the distance between the two regions increased. The results were subjected to a one-way repeated-measures ANOVA, which showed a significant main effect of conditions (*F*(9,171) = 56.094, *p* < 0.001). The post hoc analysis (Tukey's test) showed that the performance using temporal E of the Region 1 + 2 condition was significantly better than the performances under all other conditions with two frequency regions, and the performance using temporal E of the Region 1 + 3, Region 1 + 4, and Region 3 + 4 conditions was better than that under the other conditions with two frequency regions. If one frequency region was combined with another frequency region, the scores obtained from conditions combined with Frequency Region 1 would be higher than those obtained from conditions combined with other regions. For example, if the Frequency Region 2 was combined with another Frequency Region, the score of Region 1 + 2 condition was significantly higher than scores of any other combinations with Frequency Region 2, such as Region 2 + 3, Region 2 + 4, and Region 2 + 5 conditions. However, the difference between scores of conditions is not significant when Region 1 + 3 or Region 1 + 4 was compared with Region 3 + 4 and when Region 1 + 5 was compared with Region 3 + 5 or Region 4 + 5.

### 3.2. Relative Weights of the Five Frequency Regions

**Figure 3:**
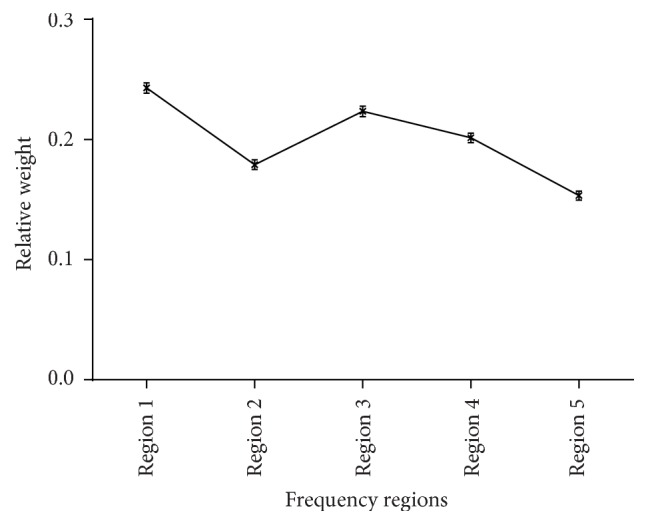
The relative weights of different frequency regions for Mandarin sentence recognition using acoustic temporal envelope. The error bars indicate standard errors.

To calculate the relative weight of the different frequency regions for Mandarin sentence recognition using acoustic temporal E, the least-squares approach described by Kasturi et al. (2002) was used. First, the strength of each region was defined to be a binary value which is either 0 or 1 depending on whether the region is presented or not. Then a linear combination of the strength of each region was applied to predict the responses of the listeners, and the weight of each region was calculated by minimizing the sum of all the squared prediction errors. The raw weights for the five regions of each listener were transformed to relative weights by summing their values and each region's weight was expressed as the raw weight divided by this sum. Therefore, the sum of the weights of the five regions was equal to 1.0. As shown in Figure 3, the mean weights of Regions 1–5 were 0.25, 0.18, 0.22, 0.20, and 0.15, respectively. The one-way ANOVA showed a significant main effect of region on weight for sentence recognition (*F*(4,76) = 60.129, *p* < 0.001). The post hoc tests (Tukey's test) showed that the relative weights differed significantly between any two frequency regions. The temporal E of Frequency Regions 1 and 3 contributed more to the intelligence of sentence recognition in Mandarin Chinese than the E cues of the other frequency regions.

## 4. Discussion

By systematically altering the stimuli presented to listeners, recognition scores with different frequency regions using acoustic E cues were recorded. Frequency-weighting functions were obtained using a least-squares approach to assess the relative contributions of temporal E cues across different frequency regions in Mandarin sentence perception. While the relative contribution of the temporal envelope across different frequency regions in English perception has been studied thoroughly [13, 15, 28], this is the first report to discuss the issue for Mandarin sentence, a tonal language.

As can be seen from Figure 2, the intelligence performance was very good when the temporal E cues of all frequency regions were presented (full region); indeed, all listeners scored perfectly. This result is consistent with previous results showing that envelope information alone is sufficient for speech intelligibility in a quiet environment [3, 29, 30]. Nevertheless, the sentence recognition correct scores were only about ~10% when the acoustic E cue from one frequency region was presented alone (Figure 1). When the acoustic E cues from any two frequency regions were presented, the performances were better than the simple sum of the scores obtained with the acoustic E cues of two corresponding frequency regions presented individually. This synergistic effect has been observed previously [11, 13, 31]. Warren et al. [11] found that the regions centered at 370 and 6,000 Hz interacted synergistically when integrated but provided little information when presented individually. Healy and Warren [31] also showed that unintelligible individual speech regions became intelligible when combined, and this effect is similar to the CI simulation results that showed a performance improvement when the number of channels increased from 1 to 2. However, Healy and Warren focused only on pair regions that had equal logarithmic separation from the frequency at 1,500 Hz. In this paper, we recorded the performance under various conditions, with all potential combinations between frequency regions, to drive the relative weight of acoustic E cues in different frequency regions.

The frequency-weighting functions indicated that the five frequency regions contributed to sentence recognition differently. Regions 1 (80–502 Hz) and 3 (1,022–1,913 Hz) were more important than the other regions. The importance of the middle-frequency range (similar to Region 3 in this study) is consistent with previous studies. The Articulation Index (AI) [32] suggested that the 1,500–2,000 Hz frequency region was most important, and Kasturi et al. [33] found that the recognition of vowels and consonants was reduced if the frequency region centered at 1,685 Hz was removed. Moreover, the mean crossover frequencies for temporal envelope speech, an indication of the frequency region providing the most information, were estimated to be 1,421 and 1,329 Hz for male and female speakers, respectively [3]. All of these results indicated that the frequency region around 1,500 Hz is important for speech recognition.

However, the relative weight of acoustic E in the low-frequency region (80–502 Hz) was highest in the present study, in contrast to the study of Ardoint et al. [15], which showed that the E information from the 1.8–7.3 kHz frequency region was more important than other regions for English recognition. Regarding the differences observed between that study and the present study, we suggest four possible reasons. First, Ardoint et al. used vowel-consonant-vowel (VCV) stimuli as test materials, while Mandarin sentences in conversational style were presented in this experiment. The context of the sentence, which is absent in the VCV stimuli, may play a role in this difference. Second, the temporal E information used by Ardoint et al. was extracted from each 2-ERB_N_-wide band and then summed in the stimuli presented to the listeners. To better model the frequency selectivity of the normal cochlea, the temporal E information presented in this paper was extracted from 30 continuous 1-ERB_N_-wide frequency bands. Third, there were obvious synergistic effects of acoustic E cues between different frequency regions in this study, especially when E cues from Frequency Region 1 were combined with any other frequency region. Thus, the temporal E cues of Frequency Region 1 (80–502 Hz) weighed most heavily here. In contrast, Ardoint et al. suggested that the performance with the E information from two frequency regions could be predicted by the performances when only one frequency region was available. Although no evident synergistic effect was observed, the sentence recognition scores with the E cues from 2 frequency regions tended to be higher if Region 4 (1,845–3,726 Hz) was selected as 1 of the 2 frequency regions [15]. Thus, their results actually showed that E cues from frequency regions above 1.8 kHz transmitted more information. Therefore, synergistic effects should have contributed to the high weight of the low-frequency region in Mandarin recognition.

Indeed, the most important difference was likely the difference in languages. As a tonal language, the recognition of tones contributes significantly to Mandarin recognition because the tonality of a monosyllable is lexically meaningful [20, 21, 34]. It is generally accepted that the tone recognition relies mainly on the variation in *F*_0_ [19, 20, 35]. Kuo et al. [36] showed that the explicit *F*_0_ cue contributed to tone recognition the most, with which listeners could consistently score >90% correct. And the temporal coding of *F*_0_ and the amplitude envelope could contribute to tone recognition more or less in the absence of explicit *F*_0_. Studies have also found a significant correlation between amplitude modulation processing and Mandarin tone recognition without explicit *F*_0_. Also, Mandarin tone recognition has been shown to improve with enhanced similarity between the amplitude and *F*_0_ contour [17, 35, 37].

Considering the essential role of *F*_0_ in tone perception and the importance of tone recognition in Mandarin sentence recognition, it seems reasonable to expect a higher weight for the low-frequency region (Region 1) for Mandarin sentence perception than nontonal English recognition. Similarly, Wong et al. [38] found that the frequency importance weight for Cantonese was inconsistent with English, due to language differences. Compared with English, the low-frequency information contributes more to Cantonese recognition, which was attributed to the tonal nature of Cantonese. Moreover, the one-third octave band (<180 Hz), which contained *F*_0_ of male speakers, weighed more than each of the four one-third octave bands between 180 and 450 Hz [38]. Furthermore, Kong and Zeng [39] found a relationship between the formant 1 (*F*_1_) frequency and the four Mandarin lexical tones. Therefore, the partial *F*_1_ in Frequency Region 1 may also contribute to tone recognition.

Knowledge of the extent to which each frequency region contributes to Mandarin sentence perception may allow us to modify the programing strategy to maximize the benefit of a cochlear implant by taking advantage of electrodes mapping to the frequency regions that weigh the most. Similar to the “normal weighting functions” for English described by Turner et al. [28], the frequency-weighting functions in this paper indicate a “normal” pattern for Mandarin perception. Based on the comparable effect of the “hole” on the performance of normal-hearing listeners and those with cochlear implants [12], knowledge of the higher weights of Frequency Regions 1 and 3 for some normal-hearing listeners here may have clinical implications for both those with cochlear implants and hearing-impaired listeners, shedding some light on the development of rehabilitation treatment for Chinese patients. The speech signals in the frequency regions with higher weights might be gained before being transmitted to the corresponding electrodes of cochlear implant. And the high weight of the Region 1 suggested the potential of the bimodal hearing [40], which would take good advantage of the speech information in the low-frequency regions, to help the cochlear implanters perform better in Mandarin speech recognition.

However, we concentrated only on the recognition of Mandarin sentences in normal listeners, and the unique frequency-weighting functions for hearing loss and cochlear implants remain unknown. Mehr et al. [41] showed that the relative weights of different regions for cochlear implant users differed from those of normal listeners. In comparison with normal-hearing listeners, Wang et al. [42] suggested that the listeners with hearing loss suffered from a lack of the ability to use spectral envelope cues for lexical tone recognition due to a reduction in frequency selectivity. Turner et al. [43] indicated that listeners with hearing impairment could not compare and integrate the temporal patterns in different frequency regions as effectively as normal hearers. Using the same speech stimuli and the region division of Turner et al. [28], Apoux and Bacon [14] suggested that a severe reduction in frequency resolution led to an increased weight of the high-frequency region. Moreover, patients with sensorial hearing loss generally suffered from reduced frequency selectivity; their frequency-weighting functions may differ from those with normal hearing. Thus, further studies are needed to address the relative importance of the different frequency regions in Chinese speakers with hearing impairment.

## 5. Conclusions

Frequency-weighting functions for temporal E information were obtained to evaluate the different contributions of various frequency regions to Mandarin sentence recognition. The results indicated that the temporal E cues of Frequency Regions 1 (80–502 Hz) and 3 (1,022–1,913 Hz) were more important than other regions. Compared with the recognition of English, the low-frequency region defined using the parameter conditions here contributed more to Mandarin sentence perception due to the tonal nature of Mandarin.

## Figures and Tables

**Table 1:** Cutoff frequency for extracting temporal envelope information.

Frequency regions	Bands	Lower frequency (Hz)	Upper frequency (Hz)
1	1	80	115
	2	115	154
	3	154	198
	4	198	246
	5	246	300
	6	300	360
	7	360	427
	8	427	502

2	9	502	585
	10	585	677
	11	677	780
	12	780	894
	13	894	1022

3	14	1022	1164
	15	1164	1322
	16	1322	1499
	17	1499	1695
	18	1695	1913

4	19	1913	2157
	20	2157	2428
	21	2428	2729
	22	2729	3066
	23	3066	3440
	24	3440	3856

5	25	3856	4321
	26	4321	4837
	27	4837	5413
	28	5413	6054
	29	6054	6767
	30	6767	7562

**Table 2:** Comparison of percent-correct scores for conditions with two adjacent frequency regions in two groups.

Conditions	Scores of Group 1	Scores of Group 2	*t*-test
Region 1 + 2	89.6 ± 5.4 (%)	87.7 ± 4.3 (%)	*p* = 0.219
Region 2 + 3	74.0 ± 5.0 (%)	77.2 ± 6.4 (%)	*p* = 0.091
Region 3 + 4	79.2 ± 6.7 (%)	79.9 ± 7.9 (%)	*p* = 0.764
Region 4 + 5	68.8 ± 9.2 (%)	69.7 ± 7.7 (%)	*p* = 0.746
